# The Prepetrous Segment of the Internal Carotid Artery as a Neglected Site of Symptomatic Atherosclerosis: A Single-Center Series

**DOI:** 10.3390/jcm13061696

**Published:** 2024-03-15

**Authors:** Marialuisa Zedde, Ilaria Grisendi, Federica Assenza, Manuela Napoli, Claudio Moratti, Lara Bonacini, Giovanna Di Cecco, Serena D’Aniello, Claudio Pavone, Giovanni Merlino, Jukka Putaala, Franco Valzania, Rosario Pascarella

**Affiliations:** 1 Neurology Unit, Stroke Unit, Azienda Unità Sanitaria Locale-IRCCS di Reggio Emilia, Viale Risorgimento 80, 42123 Reggio Emilia, Italy; grisendi.ilaria@ausl.re.it (I.G.); assenza.federica@ausl.re.it (F.A.); valzania.franco@ausl.re.it (F.V.); 2 Neuroradiology Unit, Azienda Unità Sanitaria Locale-IRCCS di Reggio Emilia, Viale Risorgimento 80, 42123 Reggio Emilia, Italy; napoli.manuela@ausl.re.it (M.N.); moratti.claudio@ausl.re.it (C.M.); bonacini.lara@ausl.re.it (L.B.); dicecco.giovanna@ausl.re.it (G.D.C.); daniello.serena@ausl.re.it (S.D.); pavone.claudio@ausl.re.it (C.P.);; 3 Clinical Neurology, Stroke Unit Department of Head, Neck and Neurosciences, Udine University Hospital, 33100 Udine, Italy; giovanni.merlino@asufc.sanita.fvg.it; 4 Departments of Neurology, Helsinki University Hospital, University of Helsinki, Haartmaninkatu 4, P.O. Box 340, 00290 Helsinki, Finland; jukka.putaala@hus.fi

**Keywords:** stroke, young, complicated atheroma, ulceration, magnetic resonance imaging (MRI), computed tomography angiography (CTA), vessel wall imaging, prepetrous internal carotid artery (ICA)

## Abstract

(1) **Background:** Non-stenotic complicated plaques are a neglected cause of stroke, in particular in young patients. Atherosclerosis has some preferential sites in extracranial arteries and the prepetrous segment of the internal carotid artery has been rarely described as site of atheroma in general and of complicated atheroma in stroke patients. The aim of this study is to describe the rate of the prepetrous internal carotid artery’s (ICA) involvement in a single-center case series of young stroke patients. (2) **Methods:** All patients < 50 years old with acute ischemic stroke admitted to a single-center Stroke Unit during two time periods (the first one from 1 January 2018 to 31 December 2019, and the second one from 1 January 2021 to 30 June 2022), were prospectively investigated as part of a screening protocol of the Searching for Explanations for Cryptogenic Stroke in the Young: Revealing the Etiology, Triggers, and Outcome (SECRETO) study [ClinicalTrials.gov ID NCT01934725], including extracranial vascular examination by using computed tomography (CT) or magnetic resonance imaging (MRI). (3) **Results:** Two out of ninety-three consecutive patients (2.15%) had a complicated atheroma in the prepetrous ICA as the cause of stroke and both CT angiography and high-resolution vessel wall MRI were applied to document the main features of positive remodeling, cap rupture, ulceration, intraplaque hemorrhage, and a transient thrombus superimposed on the atheroma. The two patients had a different evolution of healing in the first case and a persisting ulceration at 12 months in the second case. (4) **Conclusions:** The prepetrous ICA is a rarely described location of complicated atheroma in stroke patients at all ages and it represents roughly 2% of causes of acute stroke in this single-center case series in young people.

## 1. Introduction

Atherosclerosis is one of the main causes of ischemic stroke worldwide. The preferential pattern and distribution of extracranial carotid atherosclerosis has been assessed in symptomatic patients using different imaging techniques, starting from old angiographic studies until modern CT angiography (CTA) and MR angiography (MRA) studies to ultrasound examination. Most studies focused on atherosclerotic carotid stenosis in the carotid bulb, i.e., the 20 mm segment around the carotid bifurcation, but did not consider atherosclerotic carotid plaques outside the bulb. However, outside the carotid bulb, the distal internal carotid artery (ICA) is a rare site of carotid atherosclerosis in asymptomatic subjects and an even rarer site of symptomatic plaques [1]. Moreover, among the features of acute symptomatic plaques, the presence of thrombus partially adherent to the plaque surface and partially floating in the arterial lumen is one of the most significant and is associated with artery-to-artery embolism as a mechanism of ischemic lesions. The distal extracranial ICA segment, corresponding to the prepetrous segment of the ICA, is included in the C1 ICA segment in the classification of Bouthillier et al. [2] and in the previous proposal of Lasajunias et al. [3], which is mainly based on embryology. Although not different from the proximal ICA segment, the distal segment or prepetrous segment is much less prone to atherosclerosis and much more involved in other vascular diseases, e.g., dissection [4]. Therefore, in young patients, in whom carotid artery dissection is considered a more common cause of stroke than atherosclerosis, the identification of an injured prepetrous segment in the ICA of the symptomatic side is often more easily diagnosed as dissection. This single-center case series aimed to identify the prevalence of symptomatic atherosclerosis in the prepetrous ICA in a cohort of young patients admitted to a neurological Stroke Unit because of an acute ischemic stroke.

## 2. Materials and Methods

All patients <50 years old admitted to the Stroke Unit in Reggio Emilia hospital within two time periods, from 1 January 2018 to 31 December 2019 and from 1 January 2021 to 30 June 2022, were examined and prospectively screened accordingly to the protocol of the Searching for Explanations for Cryptogenic Stroke in the Young: Revealing the Etiology, Triggers, and Outcome (SECRETO) study [ClinicalTrials.gov ID NCT01934725]. The protocol of the SECRETO study [5] required, according to the best clinical practice, a study of the extracranial and intracranial vessels using CTA and/or MRA, mainly in order to exclude an arterial dissection. Among the patients screened but not enrollable in the SECRETO study because of the presence of a known cause of stroke, all neuroimaging studies, in particular CTA and MRA of the extracranial and intracranial arteries, were reviewed by two neuroradiologists to identify the presence of patients with symptomatic atherosclerosis of the prepetrous ICA. Patients were considered to have stroke on the basis of evidence of a new ischemic lesion on neuroimaging (brain MRI and/or CT) and symptomatic prepetrous atherosclerosis was defined as presence of a plaque with MRI sign of intraplaque hemorrhage or rupture and superimposed thrombus on neuroimaging studies, according to the highest degree of probability in the ASCOD classification [6]. All patients identified as having prepetrous atherosclerosis were followed-up from the clinical and neuroradiological point of view. The treatment was chosen on the basis of the decisions of the treating physician.

The enrolment time period for the present study was chosen in order to avoid the first and second pandemic waves because of the pro-thrombotic impact of SARS-CoV2 as potential confounder [7,8].

## 3. Results

**Figure 1:**
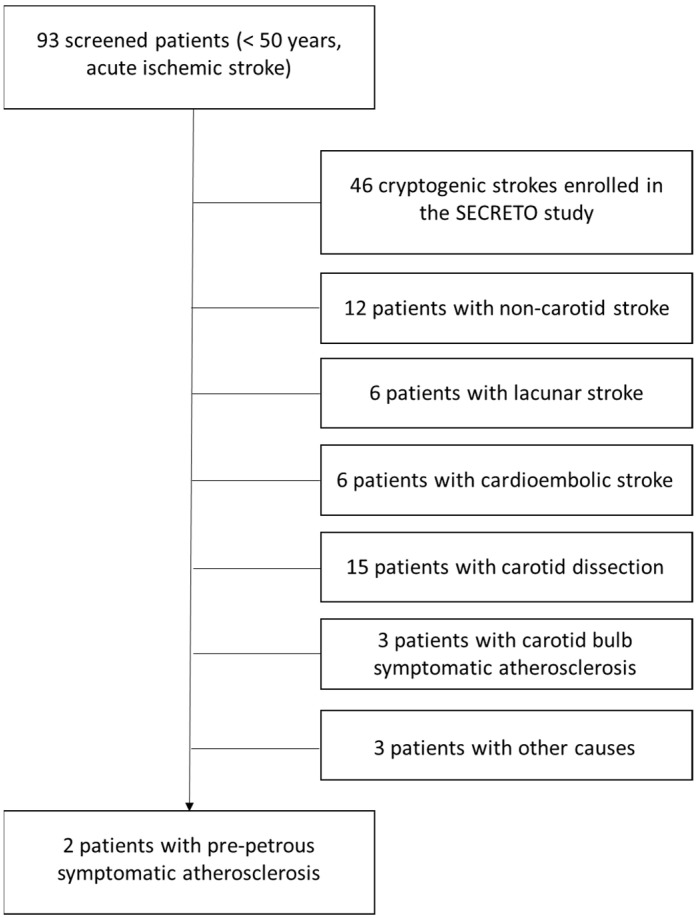
Schematic view of the selection of patients. The etiologic categories of stroke are based on the diagnostic work-up detailed in the protocol of the SECRETO study [5] and consider cardioembolic stroke as stroke from a high-risk cardioembolic source and lacunar stroke as stroke in the territory of a perforating artery due to small-vessel disease. Other stroke causes include reversible cerebral vasoconstriction syndrome (1 case), anti-phospholipid antibody syndrome (1 case), and cerebral autosomal dominant arteriopathy with subcortical infarcts and leukoencephalopathy (CADASIL (1 case). Non-carotid stroke refers to the involvement of vertebrobasilar territory.

In the selected time periods, the patients admitted to the Stroke Unit because of an acute ischemic stroke were distributed as follows in Figure 1. 

Two out of ninety-three screened patients with acute ischemic stroke < 50 years of age were found to have symptomatic atherosclerosis in the prepetrous ICA, i.e., 2.15%, which is only mildly inferior to the number of patients with symptomatic atherosclerosis in the most frequent location irrespectively of age (3/93, 3.22%). The two patients are described in detail.

### 3.1. Case 1

**Figure 2:**
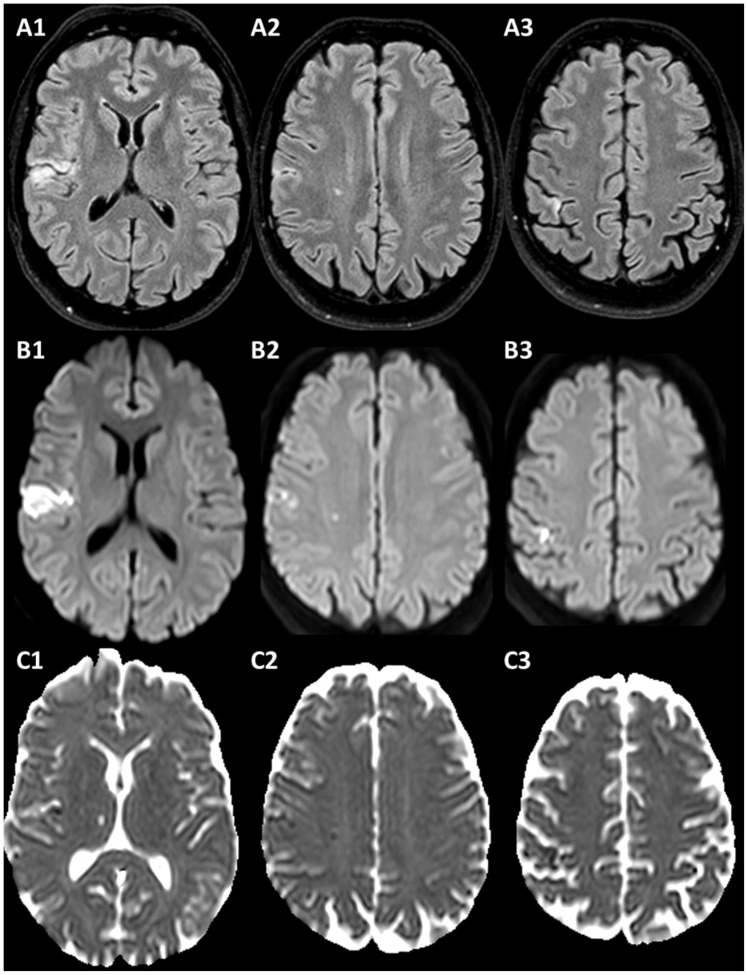
Brain MRI of Case 1. (**A1**–**A3**) are axial FLAIR slices showing multiple cortical and subcortical hyperintense areas in the right hemisphere, with corresponding DWI hyperintensity (**B1**–**B3**) and ADC decrease (**C1**–**C3**), as recent ischemic lesions.

The patient was a 49-year-old man admitted to the Stroke Unit because of the abrupt onset of left hemisensory syndrome with a non-contrast CT documentation of multiple hypodense lesions in the right frontal opercular region and in the post-central gyrus. His past medical history was unremarkable and non-treated hyperlipidemia was the only known vascular risk factor at admittance. Family history was non-significant. The patient underwent a thorough set of vascular investigations, including prolonged electrocardiographic monitoring and transthoracic and transesophageal echocardiography, without relevant findings. Laboratory tests for autoimmunity and congenital and acquired thrombophilia were normal. His blood lipid profile was as follows: total cholesterol 207 mg/dL, LDL cholesterol 166 mg/dL, HDL cholesterol 41 mg/dL, triglycerides 86 mg/dL, lipoprotein (a) 67 mg/dL (values in mmol/L are 5.5, 4.3, 1.1, 1.0, respectively). The pattern of multiple cerebral ischemic lesions was better detailed by MRI, as shown in Figure 2. 

**Figure 3:**
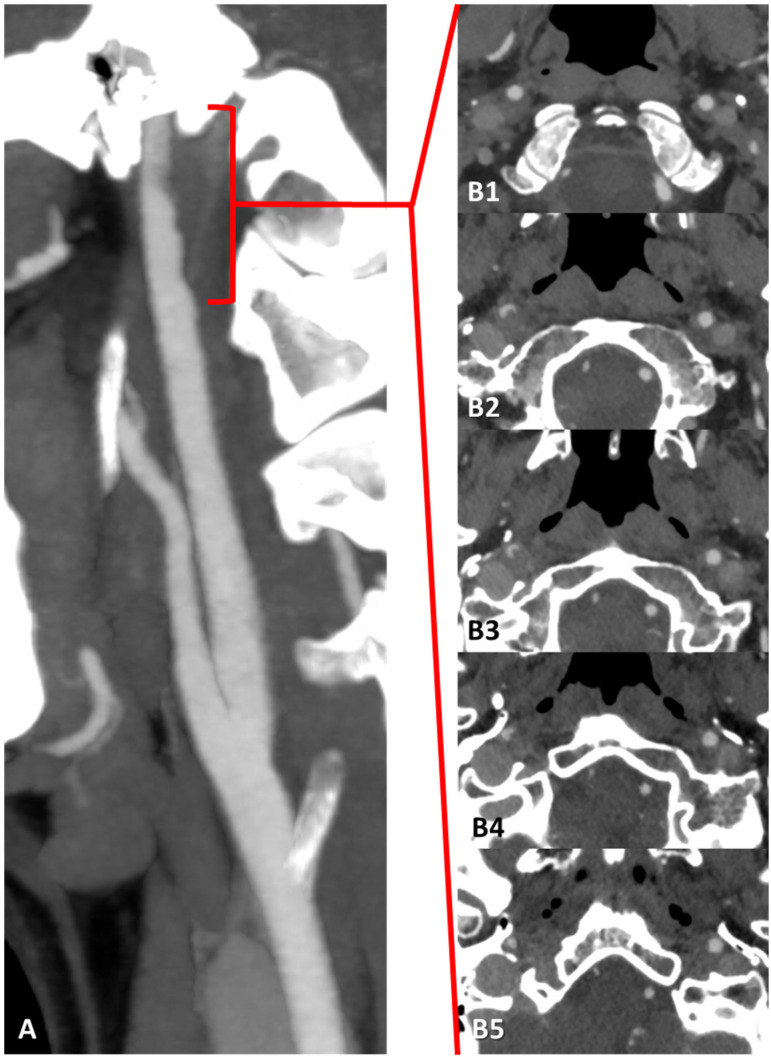
CTA in MIP/MPR reconstruction of the right ICA in oblique plane, showing an irregular luminal profile in the posterior wall in the prepetrous segment (**A**) (red square brackets). (**B1**–**B5**) are the corresponding source axial slices in a proximal-to-distal sequence, showing with better detail the hypodense structure protruding into the right ICA lumen as complicated atheroma with superimposed thrombus.

The distribution of ischemic lesions involved the territory of the right middle cerebral artery (MCA), which was patent on MRA together with the intracranial course of the ipsilateral ICA. CTA of the extracranial and intracranial arteries was performed, showing a hypodense crescent on the wall of the right prepetrous ICA with an irregular luminal surface and a small protruding component (Figure 3). The right proximal ICA showed a small hypodense plaque with a regular surface. 

**Figure 4:**
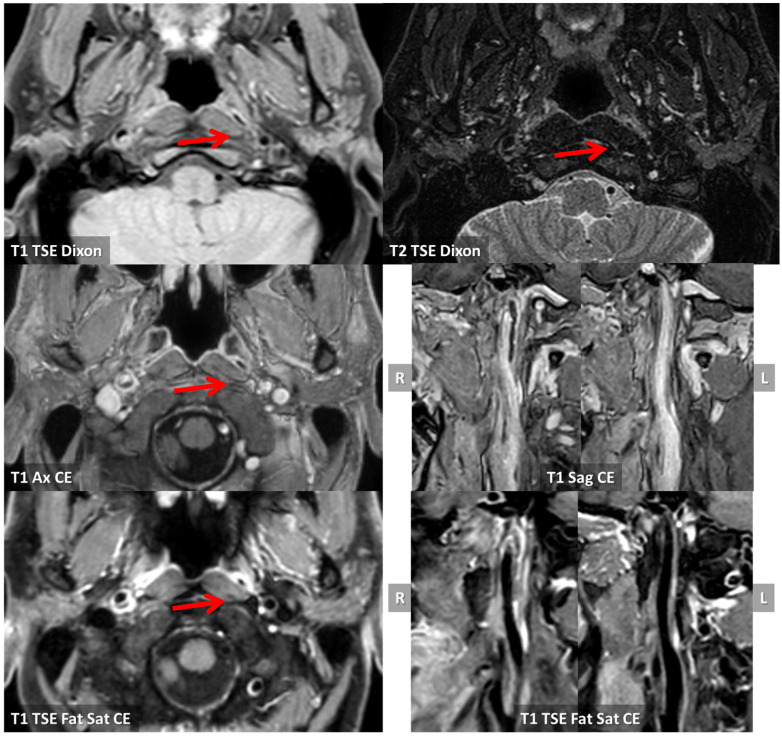
MRI with vessel wall imaging at the level of the right prepetrous ICA. In the first line, T1 and T2 TSE Dixon sequences in axial plane are imaged, showing a hyperintense luminal layer delimiting a hypointense region in the vessel wall in the posterior half (red arrows). In post-contrast T1 sequences, both in axial and sagittal planes (lines 2 and 3 of the figure), no contrast enhancement within the hypointense region is evident, but a vivid peripheral contrast enhancement was found.

In order to perform a differential diagnosis versus dissection, an MRI study with vessel wall imaging was conducted and the main findings are summarized in Figure 4. The pattern of imaging supported the diagnosis of complicated atheroma and the MRI study in Figure 4 corresponds to the B2 level of the CTA imaged in Figure 3.

Considering the neuroimaging-supported diagnosis of complicated atheroma with superimposed thrombosis in the right prepetrous ICA, antithrombotic therapy was empirically chosen using low-molecular-weight heparin (LMWH) at an anticoagulant dose and a single antiplatelet (aspirin 100 mg). Statin therapy was started with atorvastatin 80 mg/OD. 

**Figure 5:**
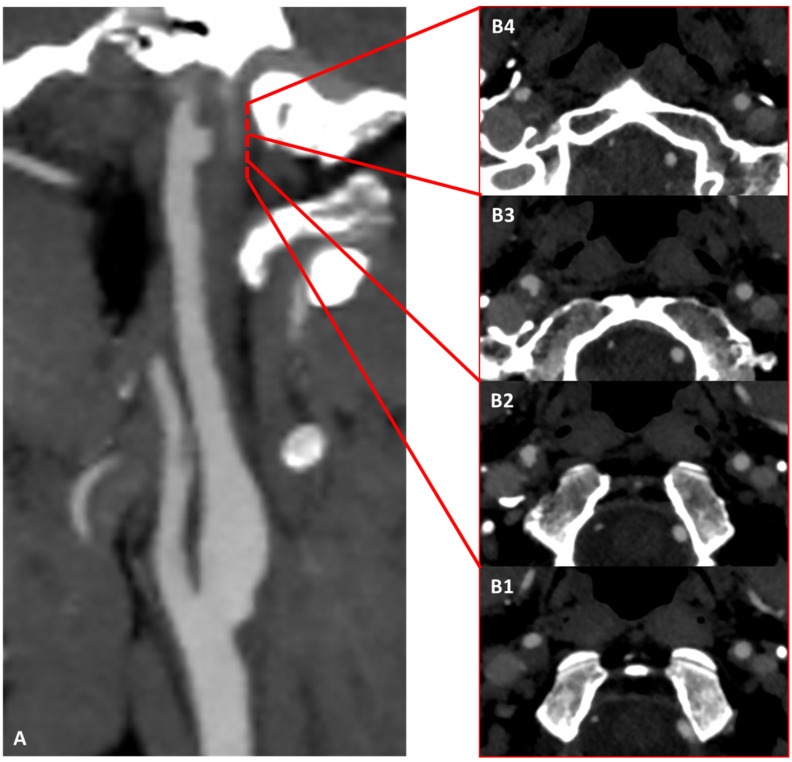
CTA in MIP/MPR reconstruction of the right ICA in oblique plane (**A**), showing an atheroma with ulceration on the posterior wall of the prepetrous segment. (**B1**–**B4**) are the corresponding source axial slices in a proximal-to-distal sequence, at a level corresponding to the intersection points of the continuous red line on the dotted red line in panel (**A**).

**Figure 6:**
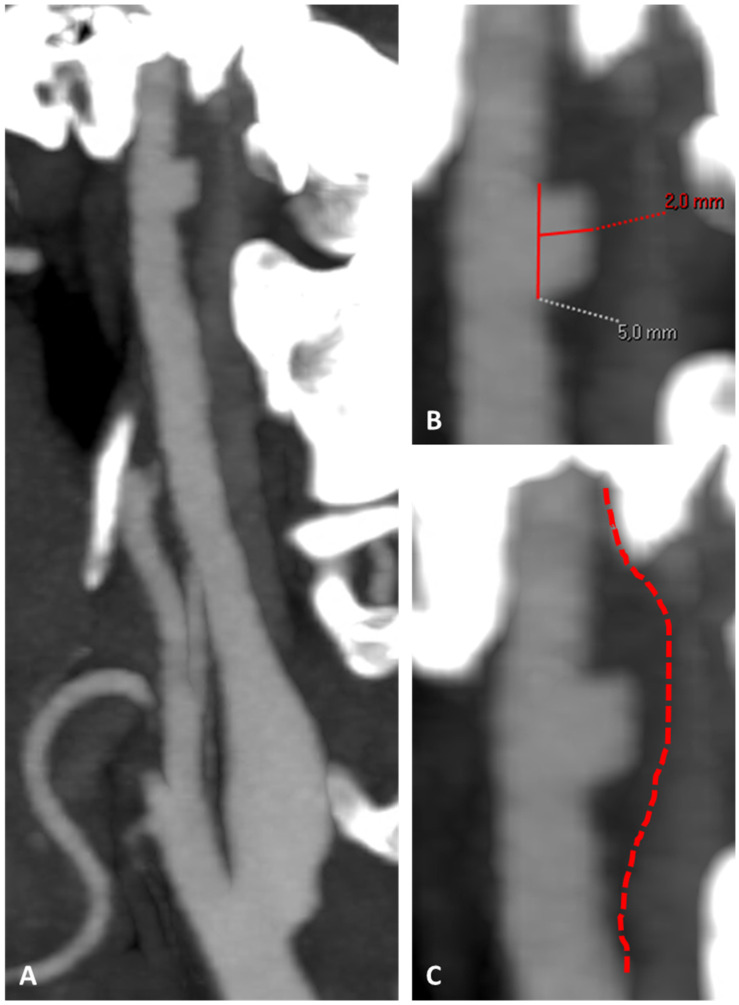
CTA in MIP/MPR reconstruction of the right ICA in oblique plane, showing atheroma with ulceration on the prepetrous segment (**A**). Panel (**B**) shows a magnified detail with measurements of the longitudinal and axial extent of the ulceration; panel (**C**) makes evident the positive remodeling (red dotted line limiting the outer border of the atheroma).

Clinical and neuroimaging follow-up was performed with an early step at 20 days from the CTA showed in Figure 3. The new CTA (Figure 5) found a resolution of the thrombus superimposed on the right prepetrous ICA atheroma, leaving the plaque ulceration well imaged (detail in Figure 6). 

**Figure 7:**
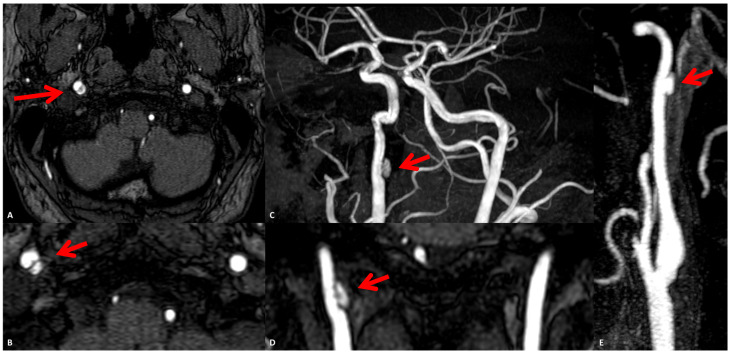
Two-month MRA. Time-of-flight (TOF) MRA source images in axial plane (**A**,**B**) showing the positive remodeling of the right prepetrous ICA at the level of the ulcerated atheroma (red arrows). MIP panoramic view TOF MRA (**C**) and MPR coronal MRA (**D**) showing the same finding with flow signal into the ulceration (red arrows). CEMRA reconstructed in MIP/MPR (**E**) confirming the same finding (red arrow).

**Figure 8:**
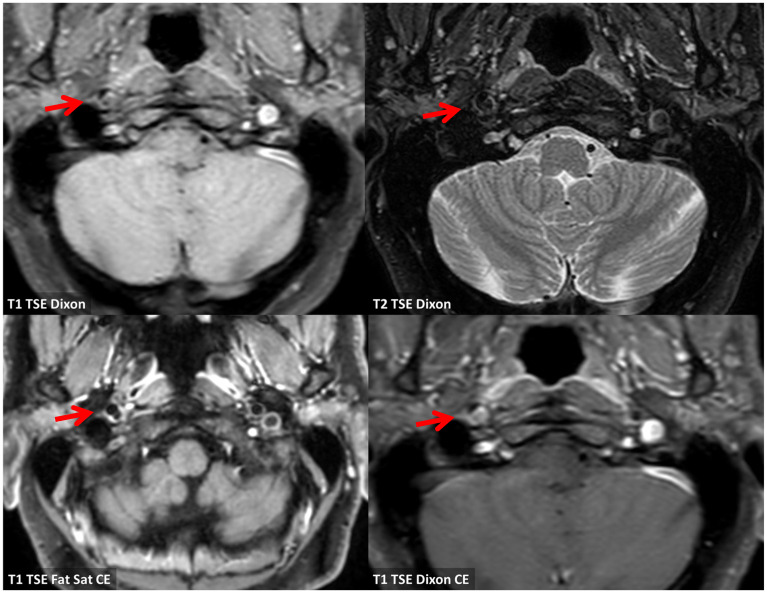
Vessel wall MRI in axial view at a level corresponding to the already described atheroma of the right ICA (red arrows). There is a focal signal abnormality on T1WI and T2WI supporting the hypothesis of a complicated plaque. In the lower row, post-contrast sequences are illustrated with focal enhancement as in cap rupture (red arrows).

At this step, LMWH was stopped and a second antiplatelet was added, continuing the antithrombotic therapy with aspirin 100 mg and clopidogrel 75 mg. No new clinical events were reported and the 2-month MRI did not show new ischemic lesions. In the same study, the presence of an ulcerated atheroma on the prepetrous ICA on the right side was confirmed (Figure 7 and Figure 8).

**Figure 9:**
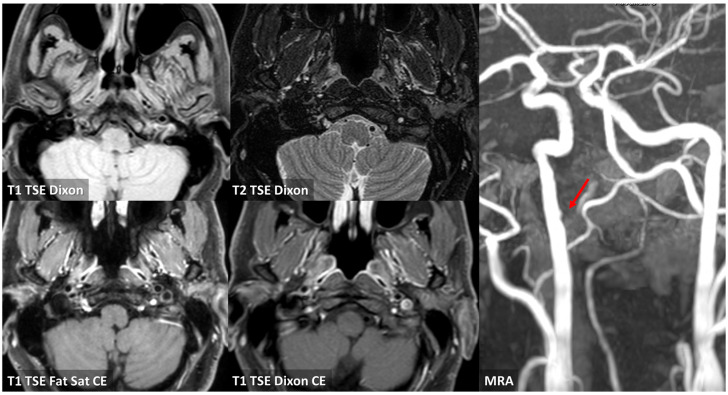
MRI with vessel wall imaging and MRA 4 months after ischemic stroke showing an almost complete healing of vessel damage in the prepetrous segment of the right ICA with only a small eccentric thickening of the posterior wall, hyperintense in T1 and T2 Dixon sequences with a peripheral contrast enhancement. The corresponding MRA shows a very mild irregularity in the luminal profile of the right ICA in the prepetrous segment (red arrow).

Dual antiplatelet therapy was prolonged until a further MRI at 4 months (Figure 9), showing a non-significant residual atheroma in the posterior wall of the right prepetrous ICA. Statin therapy was also maintained. The dose was lowered to 40 mg/die, with an LDL cholesterol target reached within one month (45 mg/dL).

Clinical and neuroimaging follow-up was prolonged up to 1 year without changes. 

### 3.2. Case 2

**Figure 10:**
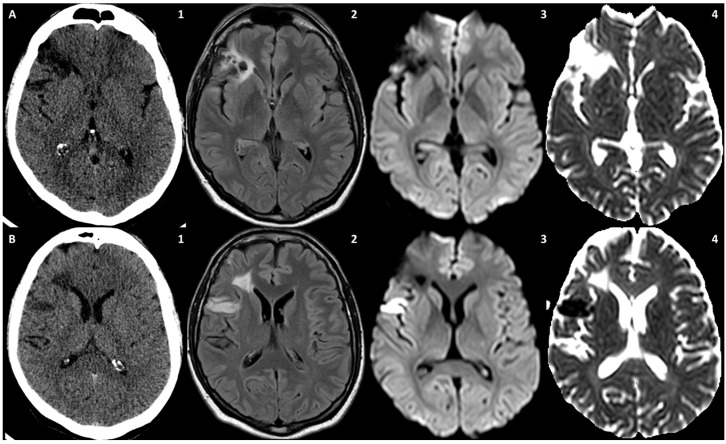
NCCT (24 h before symptom onset) (**A1** and **B1**) and brain MRI (72 h from symptom onset) (**A2**–**A4**,**B2**–**B4**)**.** (**A1**) and (**B1**) are the NCCT axial slices showing the two hypodense lesions in the right MCA territory. (**B1**) and (**B2**) are the corresponding T2-FLAIR axial slices. The anterior lesion appears mostly malacic and showed a facilitated diffusion on DWI (**A3**) and ADC (**A4**). On the contrary, the posterior lesion showed a restriction on DWI (**B3**) and ADC (**B4**), which was more recent.

A 49-year-old man with a history of active smoking, moderate alcohol consumption, and hyperlipidemia was admitted to ED because of the onset of left-sided weakness and dysarthria. At the first neurological evaluation, 24 h after the abrupt onset of the neurological deficit, the NIHSS score was 3 and no deficit was identifiable at day three from onset. An NCCT showed a densely hypodense area in the right frontal lobe and a milder hypodensity in the right opercular area (Figure 10). Both ischemic lesions are in right MCA territory and brain MRI confirmed that the most anterior one was a non-recent ischemic lesion and the opercular lesion was the recent ischemia. 

Tests for autoimmunity and acquired or inherited thrombophilia were normal, as were prolonged electrocardiographic monitoring and transthoracic echocardiography. Serum lipid levels were as follows: total cholesterol 254 mg/dL, HDL cholesterol 37 mg/dL, LDL cholesterol 179 mg/dL, triglycerides 212 mg/dL. A double antiplatelet treatment with aspirin and clopidogrel 75 mg was started together with atorvastatin 80 mg/die. 

**Figure 11:**
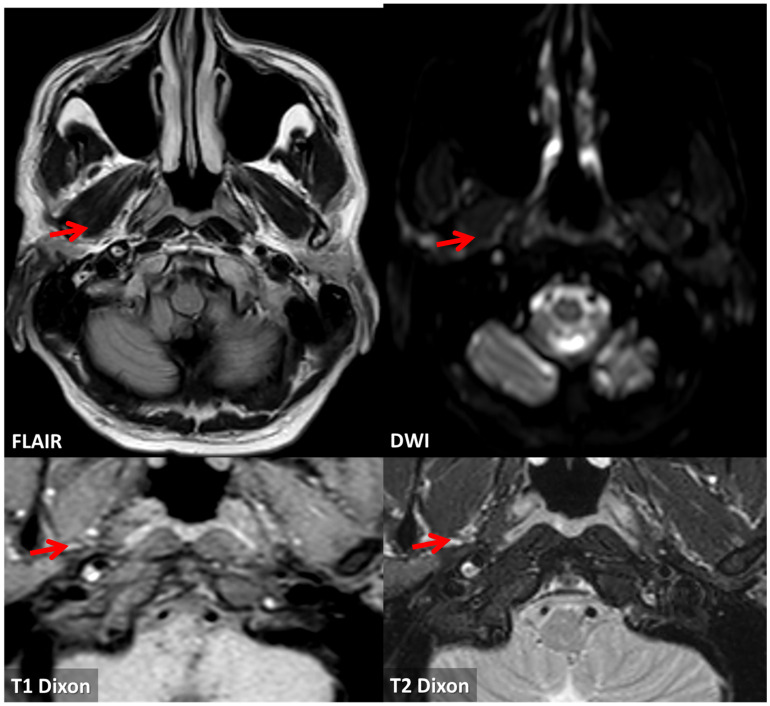
Brain MRI. On FLAIR axial slice, a hyperintense ovoidal structure is evident within the posterolateral wall of the right ICA in the prepetrous segment and a corresponding hyperintense signal is found on DWI (red arrows). The same structure is hyperintense on both T1 and T2 Dixon sequences and the signal features support the diagnostic hypothesis of complicated atheroma with intraplaque hemorrhage and positive remodeling of the corresponding segment.

**Figure 12:**
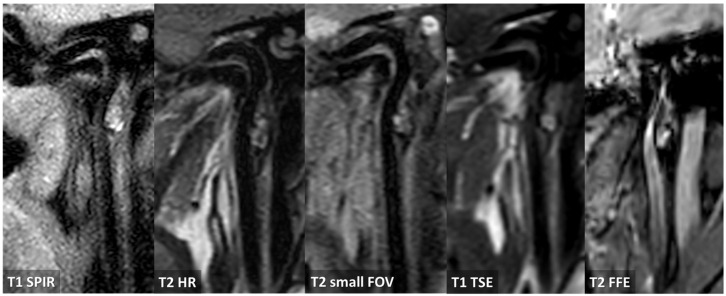
Vessel wall MRI with dedicated study of the prepetrous segment of the right ICA in oblique planes. Several sequences were used on the same segment, confirming the presence of a complicated atheroma with an intraplaque hemorrhage and a positive remodeling of the vessel wall.

During the MRI study, a complicated atheroma was found in the prepetrous segment of the right ICA, with imaging features of intraplaque hemorrhage and fibrous cap fissure (Figure 11 and Figure 12). As an incidental finding, an aneurysm of the anterior communicating artery was found. 

**Figure 13:**
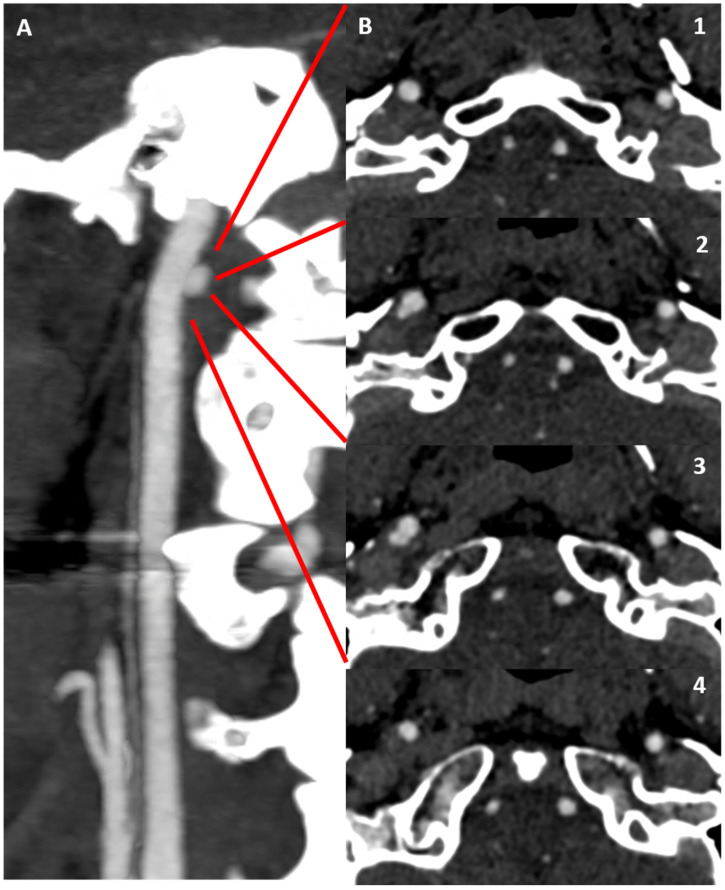
CTA focused on the right ICA. Panel (**A**) shows the MIP/MPR reconstructed oblique plane along the course of the right distal extracranial ICA with a huge ulceration of the surface of the known atheroma. In panel (**B**) are proposed the corresponding axial source images at various levels along the atheroma.

The follow-up CTA study conducted after three days showed the evolution of the complicated atheroma on the prepetrous segment of the right ICA with a plaque ulceration (Figure 13). 

No new clinical or imaging events corresponded to the change in plaque morphology discovered by CTA. 

**Figure 14:**
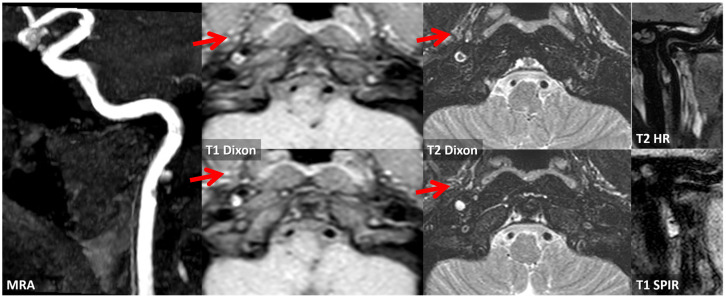
MRI study at 20 days from the first neuroimaging investigation. TOF MRA with MIP/MPR reconstruction in oblique plane confirmed the presence of an ulcerated atheroma on the prepetrous segment of the right ICA (red arrows). Both T1 and T2 Dixon axial sequences at the level of the proximal part of the atheroma and at the level of the ulceration show a hyperintense core and a linear peripheral hyperintensity. T1 HR and T2 SPIR sequences in oblique plane show the same signal features of a complicated atheroma along its longitudinal extension with positive remodeling of the involved ICA segment.

The follow-up MRI at 20 days from the first neuroimaging study did not show any changes to the known situation (Figure 14).

**Figure 15:**
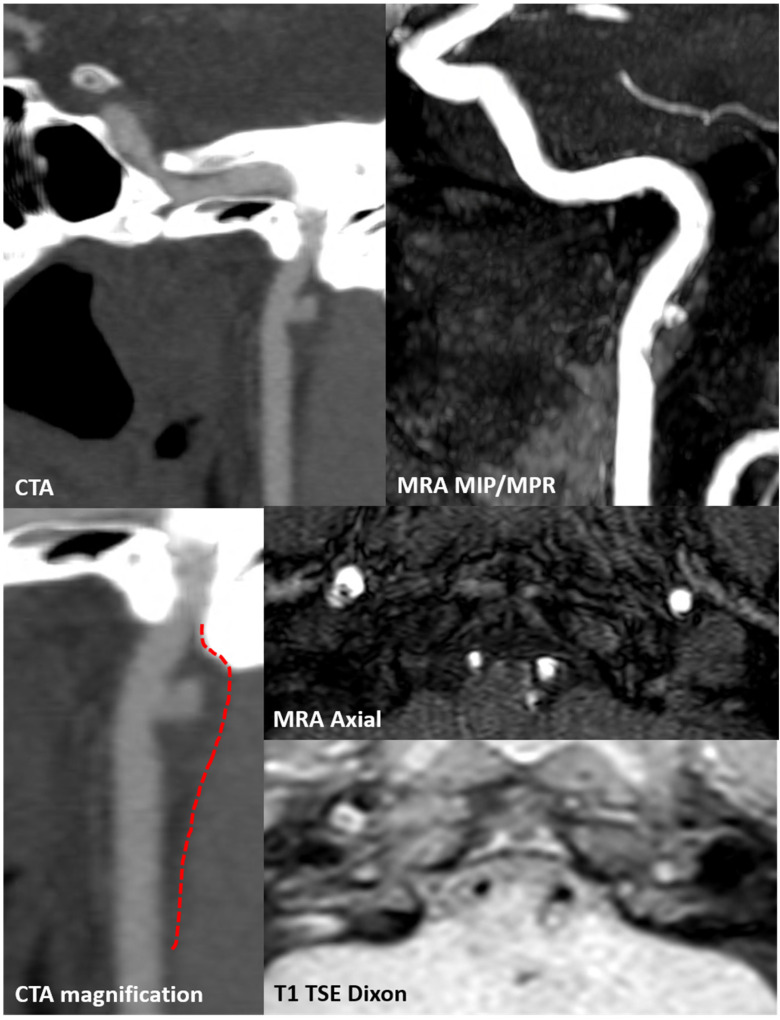
CTA and MRI study at 1 year from the first neuroimaging investigation. Both CTA and TOF MRA (MIP/MPR reconstruction on an oblique plane) showed a persisting atheroma with surface ulceration of the prepetrous segment on the right ICA. In the magnified CTA detail, the outer layer of the atheroma is outlined by a dotted red line and the positive remodeling is well evident.

Finally, both CTA and MRI at 1 year showed that the ulceration of the atheroma on the right prepetrous ICA remained unchanged (Figure 15). 

The patient did not have new vascular events at follow-up and neuroimaging studies did not show new brain lesions. Dual antiplatelet therapy was prolonged up to the CTA and MRI shown in Figure 15 and a carotid stenting was proposed and performed without complications, allowing the patient to shift toward a single antiplatelet after 30 days. High-dosage statin (atorvastatin 80 mg/die) was continued, with LDL cholesterol very close to the target (56 mg/dL). Smoking and alcohol consumption were reduced but not stopped. 

## 4. Discussion

Atherosclerosis is a relevant cause of ischemic stroke, and non-stenotic plaques might be the causes of transient ischemic attack (TIA) and stroke, although the main focus of attention has historically been directed to carotid stenosis in the carotid bulb. In fact, severe carotid artery stenosis is a well-established risk factor and approximately 11–20% of patients with either stroke or TIA have an ipsilateral carotid stenosis of >50% [9,10]. Complicated non-stenosing carotid plaques might be the cause of ischemic stroke with an embolic pattern and their role and prevalence are underestimated, as shown by the Carotid Plaque Imaging in Acute Stroke (CAPIAS) study [11]. The study enrolled patients ≥ 50 years old with unilateral carotid stroke undergoing high-resolution contrast-enhanced carotid MRI at 3T using dedicated surface coils to qualitatively evaluate the plaques at the carotid axis. The study was not designed to separate the plaques by location along the carotid artery and no information about this point was provided. The prevalence of complicated carotid plaques (fibrous cap fissure, intraplaque hemorrhage, or mural thrombus) in patients with cryptogenic stroke was significantly more frequently ipsilateral (31%) to the infarct compared with contralateral to the infarct (12%; *p* = 0.0005). A single-center study enrolling 579 stroke patients [12] showed similar results; high-risk plaque was more commonly ipsilateral versus contralateral to brain infarction in large-artery atherosclerotic (risk ratio [RR], 3.7 [95% CI, 2.2–6.1]), cryptogenic (RR, 2.1 [95% CI, 1.4–3.1]), and cardioembolic strokes (RR, 1.7 [95% CI, 1.1–2.4]). Moreover, after accounting for ipsilateral high-risk plaque, 88 (15.2%) patients were reclassified: 38 (22.6%) cardioembolic to multiple potential etiologies, 6 (8.5%) lacunar to multiple, 3 (15.8%) other determined cause to multiple, and 41 (20.8%) cryptogenic to large-artery atherosclerosis. This last issue is particularly relevant in young patients, where the category of cryptogenic embolic stroke has received attention from the therapeutical point of view rather than from the diagnostic point of view, with recent criticism of the concept of Embolic Stroke of Undetermined Origin (ESUS) [13]. 

In this context, the distal ICA is a rare location for atherosclerosis in general. It has not been clearly reported in neuroimaging studies on symptomatic patients. The main limitation of previous studies is that ultrasound techniques are the most commonly used imaging strategy in asymptomatic subjects and the distal ICA is not well imaged by ultrasound, so the main focus of these studies was the most prevalent location of atherosclerosis, i.e., the carotid bulb [14]. There is one study in an asymptomatic elderly cohort performed using MRI with vessel wall imaging [15]. The advantages of MRI are not only the spatial coverage of the entire ICA course, including the prepetrous segment, but also the possibility to estimate plaque composition and to identify unstable plaque, characterized by fibrous cap rupture, intraplaque hemorrhage, or a large lipid-rich necrotic core [16,17,18,19,20,21,22,23]. Three-dimensional (3D) MRI techniques have the following advantages compared to 2D MRI techniques: fast imaging, high isotropic spatial resolution (0.6–0.8 mm), and large longitudinal coverage (>150 mm) [24]. Representative 3D MR vessel wall imaging sequences include MERGE (motion-sensitized driven-equilibrium prepared rapid gradient echo) [25], SNAP (simultaneous non-contrast angiography and intraplaque hemorrhage) [26], and VISTA (volumetric isotropic TSE acquisition) [27]. Cai et al. [14] applied 3D multicontrast MR vessel wall imaging (MERGE and SNAP) on a 3.0T scanner to investigate the characteristics of the morphology, composition, and distribution of carotid artery atherosclerotic plaques in an asymptomatic elderly population (age ≥ 60 years). Longitudinal coverage included the distal extracranial ICA and the authors reviewed the MRI studies dividing the carotid artery into five segments: distal internal carotid artery (D-ICA); proximal ICA (P-ICA); carotid bulb (CB); distal common carotid artery (DCCA); proximal CCA (P-CCA). They examined 140 subjects, 63 (45.0%) males, mean age 72.1 ± 5.7 years old, 65 (47.1%) with arterial hypertension, 26 (19.0%) with diabetes, and 16 (11.6%) smokers. Eighty-seven (62.1%) had carotid atherosclerotic plaque and the prevalence of calcification, a lipid-rich necrotic core, intraplaque hemorrhage, and high-risk plaque was 26.4%, 45.0%, 7.9%, and 12.1%, respectively. In this population, atherosclerotic plaques were mostly found in the CB segment (33.9%), followed by PICA (13.6%), P-CCA (11.1%), D-CCA (4.6%), and D-ICA (3.6%). The D-ICA segment was the least involved, ranging from 2.9 in the left ICA to 4.3% in the right ICA. A previous study proved that atherosclerotic plaques develop largely in regions with low wall shear stress, such as the carotid bulb [28], but other sites have not been systematically investigated using modern techniques with large longitudinal coverage. Thus, the confirmed prevalence of the prepetrous ICA localization of atherosclerosis is low in patients > 60 years old with vascular risk factors and it is expected to be lower in young patients, even those with ischemic stroke. Another study which partially addressed the issue of distal ICA plaques and their management [29] did not consider the prepetrous segment of the ICA, as the median distance (mm) (IQR) of stenosis from the common carotid artery bifurcation is 20.4 (20.1–21.4). In our single-center series of young patients with acute ischemic stroke, 2/93 (2.15%) patients had symptomatic complicated plaques in the prepetrous ICA. The relevance of this finding was confirmed by neuroimaging features of complicated plaque (Figure 4, Figure 13 and Figure 14). 

In fact, the so-called vulnerable carotid plaque has several neuroimaging markers, which were recently standardized using different techniques, all focused mainly on the most frequent location of atherosclerosis (the carotid bulb). MRI is able to image plaque composition and surface in detail, identifying intraplaque hemorrhage, a lipidic or necrotic core, and surface fragmentation with or without superimposed thrombus. Thus, imaging technologies that better capture details of plaque morphology, such as the presence of a lipid-rich necrotic core (LRNC), the thickness and eventual rupture of an FC, the presence of IPH, plaque neovascularization, and calcifications, are important for improving risk prediction, selection of patients for intervention, and ultimately also for the development of preventive plaque-stabilizing pharmacotherapy. The recently published results of the CAPIAS trial showed that IPH is more prevalent on the symptomatic side in patients with carotid plaques and <50% stenosis [11]. The morphology of the luminal surface was one of the first features identified in the NASCET trial, and its alteration, in particular ulceration, was associated with an increased risk of cerebrovascular events [30]. The luminal surface of carotid plaques can be classified as smooth, irregular, or ulcerated [31]. An irregular surface indicates the presence of small alterations of the luminal surface on the luminal profile of the plaque, as in our two patients with an evolution in plaque ulceration (Figure 5, Figure 6, Figure 7, Figure 14 and Figure 15). Plaque ulceration has been defined as “an intimal defect larger than 1 mm in width, exposing the necrotic core of the atheromatous plaque” [32]. MR time-of-flight (TOF) has a suboptimal performance in the detection of ulcerations because it is prone to the saturation of slowly flowing or recirculating blood protons that can affect the signal within an ulcer crater [33], whereas contrast-enhanced magnetic resonance angiography has been demonstrated to be a sensitive technique for their identifications [34]. Intraplaque hemorrhage (IPH) is considered one of the most important features of carotid artery plaque vulnerability. Several studies have found a statistically significant association between the presence of IPH and stroke, and more recently, two important meta-analyses [35,36] have demonstrated that patients with IPH have a greater risk of stroke even in asymptomatic patients [35]. Currently, it is possible to detect IPH with MRI and CT. Because of the sensitivity of MRI in detecting blood products, some authors suggest that MRI is the best modality for the detection of IPH [37], and a recent statement published by the Carotid Imaging Consensus Group has strengthened this position [38]. The detection of IPH using CT is more complex than MRI, and there is no consensus about the use of CT for the detection of this feature. It has been suggested that the low attenuation values in carotid plaque (<30 or 25 HU) are associated with the presence of IPH [39,40,41]. The fibrous cap (FC) is a layer of connective tissue that separates the plaque from the vessel lumen. Vulnerable plaques are characterized by the presence of a thin FC or ruptured FC [42]. The fissuring or rupture of the FC exposes the LRNC to luminal blood, activating the thromboembolic cascade. On MRI, a normal FC is characterized by the presence of a juxtaluminal band of low signal on TOF MR images and/or a hyperintense juxtaluminal region on contrast-enhanced T1w images. A thin FC is present when this band of low signal on TOF or the hyperintense region on CE-T1w is not visible or when the juxtaluminal hyperintense region on CE-T1w MRI is interrupted. Using MRI, the ruptured FC is identified by (1) the absence of the juxtaluminal band of low signal and (2) the presence of a bright gray region adjacent to the lumen, corresponding to plaque hemorrhage and/or mural thrombus. 

Moreover, the identification of atherosclerosis as a cause of a transient ischemic attack (TIA) and minor stroke is of paramount importance for an adequate secondary prevention in young people too, given that atherosclerosis is associated with the highest risk of stroke recurrence and vascular diseases in long-term follow-up [43]. Indeed, this subset of patients has a much higher risk of major vascular events within 5 years than do those without atherosclerosis: 7.7% (95% CI 6.3–9.2; 101 events) in patients categorized with grade A0 atherosclerosis according to the ASCOD classification [6], 19.8% (17.4–22.4; 189 events) in those with grade A1 or A2, and 13.8% (11.8–16.0; 144 events) in patients with grade A3 [44]. In this study, no differentiation between proximal or distal ICA location of atherosclerosis was proposed and the exact location of atherosclerosis, apart the differentiation between extracranial and intracranial atherosclerosis, was outside the aims of the TIA Registry. 

Finally, the treatment of non-stenosing complicated plaques symptomatic for ischemic stroke has not been fully defined and there is no evidence-based set of recommendations or consensus. The same consideration applies to free-floating thrombus [45] superimposed on a carotid [46] or vertebral [47] plaque. In particular in Case 1, the presence of a thrombus superimposed on a complicated plaque drove the empirical choice towards LMWH at an anticoagulant dose, shifting to antiplatelets after documenting a resolution of thrombus and, at follow-up, even a resolution of ulceration, leaving a very small and non-significant atheroma. In Case 2, the choice was towards a double antiplatelet therapy because no sure documentation of protruding thrombi was provided, but a quick evolution of a persisting surface ulceration was the reason for proposing carotid stenting after 12 months of follow-up. Both patients had no recurrence of stroke/TIA, although the evolution of the atheroma was different. The limited attention paid to the prepetrous ICA as a site of symptomatic atheromatous pathology does not currently allow us to consider the proximal or distal site in the C1 segment of the ICA as characterized by different characteristics of atheroma and differences in aspects of vascular imaging, if not due to the lower frequency in the prepetrosal segment, then probably for hemodynamic reasons. Likewise, the information that is currently available does not allow us to speculate on a different treatment modality, on top of the lack of surgical accessibility. As underlined by the two cases presented, the prepetrous segment of the ICA can be the site of atheroma and also of symptomatic complicated atheroma, the treatment of which is however devoid of evidence-based strategies in the same way as described for non-stenosing atheroma of the proximal ICA.

## 5. Conclusions

Atherosclerosis is a major cause of ischemic stroke, and it should be looked for in young patients too. Unusual sites of symptomatic complicated atherosclerosis include the prepetrous segment of the ICA and can be imaged using CTA- and MRI-based techniques. These techniques are useful for a differential diagnosis versus other vascular diseases (e.g., dissection). How to use these findings to guide therapeutic decisions is still unknown.

## Data Availability

The data presented in this study are available on request from the corresponding author. The data are not publicly available due to privacy.
